# α-Mangostin-encapsulated PLGA nanoparticles inhibit pancreatic carcinogenesis by targeting cancer stem cells in human, and transgenic (Kras^G12D^, and Kras^G12D^/tp53R270H) mice

**DOI:** 10.1038/srep32743

**Published:** 2016-09-14

**Authors:** Raj Kumar Verma, Wei Yu, Anju Shrivastava, Sharmila Shankar, Rakesh K. Srivastava

**Affiliations:** 1 Kansas City VA Medical Center, 4801 Linwood Boulevard, Kansas City, MO, 66128, USA; 2 St. Joseph’s Hospital and Medical Center, Phoenix, AZ, 85013, USA; 3 Department of Pathology and Laboratory Medicine, University of Missouri Kansas City, MO, USA; 4 Department of Pharmaceutical Sciences, University of Missouri-Kansas City, Kansas City, MO 64108, USA

## Abstract

Activation of sonic hedgehog (Shh) in cancer stem cell (CSC) has been demonstrated with aggressiveness of pancreatic cancer. In order to enhance the biological activity of α-mangostin, we formulated mangostin-encapsulated PLGA nanoparticles (Mang-NPs) and examined the molecular mechanisms by which they inhibit human and KC mice (Pdx^Cre^;LSL-Kras^G12D^) pancreatic CSC characteristics *in vitro*, and pancreatic carcinogenesis in KPC (Pdx^Cre^;LSLKras^G12D^;LSL-Trp53^R172H^) mice. Mang-NPs inhibited human and Kras^G12D^ mice pancreatic CSC characteristics *in vitro*. Mang-NPs also inhibited EMT by up-regulating E-cadherin and inhibiting N-cadherin and transcription factors Slug, and pluripotency maintaining factors Nanog, c-Myc, and Oct4. Furthermore, Mang-NPs inhibited the components of Shh pathway and Gli targets. *In vivo*, Mang-NPs inhibited the progression of pancreatic intraneoplasia to pancreatic ductal adenocarcinoma and liver metastasis in KPC mice. The inhibitory effects of Mang-NPs on carcinogenesis in KPC mice were associated with downregulation of pluripotency maintaining factors (c-Myc, Nanog and Oct4), stem cell markers (CD24 and CD133), components of Shh pathway (Gli1, Gli2, Patched1/2, and Smoothened), Gli targets (Bcl-2, XIAP and Cyclin D1), and EMT markers and transcription factors (N-cadherin, Slug, Snail and Zeb1), and upregulation of E-cadherin. Overall, our data suggest that Mang-NPs can inhibit pancreatic cancer growth, development and metastasis by targeting Shh pathway.

Pancreatic cancer is the fourth leading cause of cancer-related deaths in US, with a 5-year survival rate of approximately 6%^1^. Most of the pancreatic cancer is already metastasized to distant organs by the time it is detected; therefore disease is untreatable at such a late stage. Cancer stem cells (CSCs)/tumor initiating cells (TICs) have been found to be the cause of cancer initiation, progression, metastasis, and drug resistance^2^. Several studies using genetically engineered mouse models have demonstrated the aberrant activation of signaling pathways in pancreatic cancer^3^. One such pathway is sonic hedgehog (Shh) pathway which is highly activated in pancreatic cancer and its expression is correlated with poor prognosis^4^,^5^. Therefore, there is an urgent need to target CSCs by developing strategies which inhibit the population of CSCs and also those pathways which are highly activated.

The development of natural product-based compounds can be an attractive strategy for the treatment and prevention of pancreatic cancer because they are non-toxic, and inhibit pluripotency and self-renewal capacity of CSCs by targeting multiple pathways^2^. The α-mangostin is derived from the plant mangosteen (*Garcinia mangostana*) which have originated in the Sunda Islands and the Moluccas of Indonesia^6^. Mangosteen contains xanthonoids, such as α-mangostin, and other phytochemicals^7^. It grows mainly in Southeast Asia, and also in South America^8^. It possesses antioxidant, antimicrobial, anticancer, and anti-inflammatory properties^9^,^10^,^11^,^12^. α-Mangostin suppresses EMT and lipopolysaccharide-induced invasion by inhibiting matrix metalloproteinase-2/9 and increasing E-cadherin expression through extracellular signal-regulated kinase signaling in pancreatic cancer cells^13^. It inhibits mammalian DNA polymerase and topoisomerase activities in colon cancer^14^. The beneficial effects of α-mangostin suggest that it can be further developed for the treatment and prevention of cancer.

In spite of these studies, the molecular mechanisms by which it inhibits pancreatic cancer growth by targeting CSCs have never been examined. α-Mangostin is safe and well-tolerated. Furthermore, we have recently demonstrated that α-mangostin can inhibit pancreatic CSCs characteristics by inhibiting Shh pathway i.e. disrupt Gli-DNA binding activity^15^. However, the *in vivo* application of α-mangostin is limited due to its hydrophobic nature, poor aqueous solubility and stability, and thus low bioavailability and accumulation in the target organs. To overcome this limitation, we have encapsulated α-mangostin into the core of poly (D, L-lactic-co-glycolic acid) (PLGA) nanoparticles (Mang-NPs).

Shh is a member of the Hedgehog (Hh) family of secreted signaling proteins which play diverse functions during vertebrate development and in tissue homeostasis^16^,^17^. The binding of Shh to Patched (Ptch) results in loss of Ptch activity and consequent phosphorylation and posttranscriptional stabilization of Smoothened (Smo), leading to activation of Gli transcription factor^18^,^19^. Gli activation via Smo can occur either by Hh protein stimulation or through loss of Ptch activity. We and others have demonstrated that Shh pathway is constitutively active in pancreatic cancer and inhibition of Smoothened or Gli transcription can suppress the ability of pancreatic CSCs to proliferate and self-renew^20^,^21^,^22^,^23^,^24^,^25^.

The main objective of this paper is to examine the molecular mechanisms by which Mang-NPs inhibit human and KC (Pdx^Cre^;LSL-Kras^G12D^) mice pancreatic cancer stem cell (Pan CSC) characteristics *in vitro* and tumor growth, development and metastasis in KPC (Pdx^Cre^;LSLKras^G12D^;LSL-Trp53^R172H^) mice. Mang-NPs suppresses pancreatic CSC characteristics by modulating genes involved in cell proliferation, self-renewal, pluripotency, cell cycle, apoptosis and epithelial mesenchymal transition (EMT), and also inhibit pancreatic cancer growth, development and metastasis in KPC mice by targeting CSCs. In conclusion, Mang-NPs can be used as a potential chemotherapeutic agent for the treatment and prevention of pancreatic cancer.

## Materials and Methods

### Reagents

Antibodies against CD24, CD133, c-Myc, Nanog, Oct4, Gli1, Gli2, Patched-1, Patched-2, Smoothened, Bcl2, XIAP, Cyclin D2, E-Cadherin, N-cadherin, Slug, Snail, Zeb1 and β-actin were obtained from Cell Signaling Technology (Danvers, MA). All other chemicals were purchased from Fisher Scientific (Suwanee, GA) and Sigma-Aldrich (St. Louis, MO).

### Production and characterization of α-mangostin encapsulated PLGA nanoparticles

We have produced Mang-NPs as we described earlier^26^. In brief, PLGA (50:50 PLGA, 14000–16000 MW, Sigma-Aldrich) nanoparticles (NPs) encapsulating α-mangostin (Mang) were prepared using a double emulsion-solvent evaporation method^26^. α-mangostin was purchased from the LKT (St. Paul, MN).

### Particle size and zeta potential analysis

Freeze-dried nanoparticles were suspended in deionized water. The mean particle diameter and width (polydispersity index) were determined by photon correlation spectroscopy using a Zetasizer 3000^26^. The particle charge was quantified as zeta potential by laser Doppler anemometry using the Zetasizer.

### Cell culture

We have previously described the isolation and characterization of human and Kras^G12D^ mouse pancreatic CSCs (CD133^+^/CD44^+^/CD24^+^/ESA^+^)^20^,^21^,^22^. Pancreatic CSCs were cultured in stem cell growth medium with 1% N2 Supplement (Invitrogen), 2% B27 Supplement (Invitrogen), 20 ng/ml human platelet growth factor (Sigma-Aldrich), 100 ng/ml epidermal growth factor (Invitrogen) and 1% antibiotic-antimycotic (Invitrogen) at 37 °C in a humidified atmosphere of 95% air and 5% CO_2_. Pancreatic cancer cell lines (AsPC-1, MIA PaCa-2, and PANC-1) were purchased from ATCC.

### Generation of KC (pdx^Cre^;LSL-Kras^G12D^), and KPC (pdx^Cre^;LSLKras^G12D^;LSL-Trp53^R172H^) mice

Animal procedures were approved by the Institutional Animal Care and Use Committee (IACUC) at Kansas City VA Medical Center, and were conducted in accordance with the Guide for the Care and Use of Laboratory Animals issued by the National Institutes of Health. Pdx1-Cre mice (generated by Dr. Lowy, University of Cincinnati) were purchased from the Jackson laboratory (Bar Harbor, Maine). LSL-Trp53R172H and LSL-KrasG12D/+ mice were obtained from MMHCC, NCI/NIH. The KC (Pdx^Cre^;LSL-Kras^G12D^) and KPC (Pdx^Cre^;LSLKras^G12D^;LSL-Trp53^R172H^) mice were generated as we and others have described earlier^27^,^28^,^29^. All of these genetically engineered mice were bred and genotyped for the presence of Kras, p53, and Cre^27^. Six-week-old breeding pairs of genetically engineered mice, including transgenic Pdx1-Cre, LSLTrp53 R172H, and LSL-KrasG12D mice were used for breeding. To produce compounded transgenic KPC mice, the double transgenic LSLKras^G12D/+^ -LSL-Trp53^R172/+^ mice were first generated, and then further mated with heterozygous Pdx1-Cre transgenic mice^30^. Pancreatic CSCs were isolated from the KC mice as we described elsewhere^28^. Mang-NPs were administrated intraperitoneally into KPC mice (about 4 weeks old males and females) once per day, five days per week for about 10 weeks.

### Lentiviral particle production and Nanog shRNA transduction

Nanog shRNA plasmids targeting 4 sites were obtained from Open Biosystems, Huntsville, AL) and cloned into TRIPZ vector. Lentiviral production and transduction were performed as we described elsewhere^26^.

### Cell viability and apoptosis assays

Cells (1.5 × 10^4^) were incubated with or without Mang-NPs (0–10 μM) in culture medium for 48 or 72 h. Cell proliferation was determined by trypan blue assay as described previously^26^. The apoptosis was determined by TUNEL assay.

### Tumor spheroid assay

For spheroid forming assay, cells were plated in six-well ultralow attachment plates (Corning Inc., Corning, NY) at a density of 1,000 cells/ml in stem cell growth medium as described above. Human pancreatic CSCs were treated with Mang-NPs (0–10 μM) to obtain primary spheroids. Spheroids were collected after 7 days and dissociated with Accutase (Innovative Cell Technologies, Inc.). The CSCs obtained from dissociation of spheroids were counted. At the end of incubation period, spheroids were collected, reseeded and treated with Mang-NPs for another week to obtain secondary spheroids. Secondary spheroids were collected, reseeded and treated with Mang-NPs for another week to obtain tertiary spheroids. Cell viability in spheroids was measured by trypan blue assay at the end of 7, 14 and 21 days.

### Motility assay

Scratch migration assay was performed as we described elsewhere^26^. In brief, a confluent monolayer of cells was established and then a scratch is made through the monolayer, using a standard 200 μl plastic pipette tip, which gives rise to an *in vitro* wound, washed twice with PBS, and replaced in media with or without Mang-NPs. CSC migrate into the scratch area as single cell from the confluent sides. The width of the scratch gap is viewed under the microscope in four separate areas each day until the gap is completely filled in the untreated control wells. Three replicate wells from a 6-well plate were used for each experimental condition.

### Transwell migration and invasion assays

Transwell migration and invasion assays were performed as we described elsewhere^26^. For transwell migration assay, 1 × 10^5^ CSCs were plated in the top chamber onto the non-coated membrane (24-well insert; pore size, 8 mm; Corning Inc.) and allowed to migrate in the lower chamber towards serum-containing medium. After 24 h of incubation, cells were fixed with methanol, stained with crystal violet and counted.

For invasion assay, 1 × 10^5^ cells were plated on top of a layer of Matrigel in Transwell chambers (BD Biosciences). Pancreatic CSCs were plated in medium without serum or growth factors, and medium supplemented with serum was used as a chemoattractant in the lower chamber. After 48 h of incubation, cells that invaded to the bottom chamber were fixed with methanol, stained with crystal violet and counted.

### Western blot analysis

Western blot analysis was performed as we described elsewhere^29^.

### Quantitative real-time PCR

Total RNA was isolated using an RNeasy Mini Kit (Qiagen, Valencia, CA) and q-RT-PCR was performed as described elsewhere^29^. Briefly, cDNA was synthesized using a high capacity cDNA reverse transcription kit (Applied Biosystems). Primers specific for each of the signaling molecules were designed using NCBI/Primer-BLAST and used to generate the PCR products. For the quantification of gene amplification, Real-time PCR was performed using an ABI 7300 Sequence Detection System in the presence of SYBR- Green.

### Gli reporter assay

Gli reporter activity was measured as we described elsewhere^25^,^31^. In brief, we have used a construct containing cop-GFP and luciferase genes downstream of Gli-response element (pGreen Fire1-4xGli-mCMV-EF1-Neo). Pancreatic CSCs and cell lines were transduced with lentiviral particles, and stable cells were selected. For transcription assay, pancreatic CSCs or cancer cell lines (10–40 × 10^3^ cells per well) were seeded in 12-well plates and treated with or without Mang-NPs (0–10 μM) for up to 48 h. After incubation, cells were analyzed for luciferase reporter activity (Promega Corp., Madison, WI).

### Immunofluorescence

Pancreatic CSCs and cell lines were grown in 12-well plates (Beckton Dickinson, Bedford, MA) and treated with or without a mixture of coumarin-6 containing Mang-NPs (5 μM) and Hoechst33342 (1 mg/ml) for various time points (0–24 hrs). After washing with PBS, stained cells were visualized under a fluorescent microscope.

### Statistical analysis

The mean and SD were calculated for each experimental group. Differences between groups were analyzed by one or two way ANOVA, followed by Bonferoni’s multiple comparison tests using PRISM statistical analysis software (GrafPad Software, Inc., San Diego, CA). Significant differences among groups were calculated at P < 0.05.

## Results

### Formulation and uptake of α-mangostin encapsulated PLGA nanoparticles (Mang-NPs)

**Figure 1:**
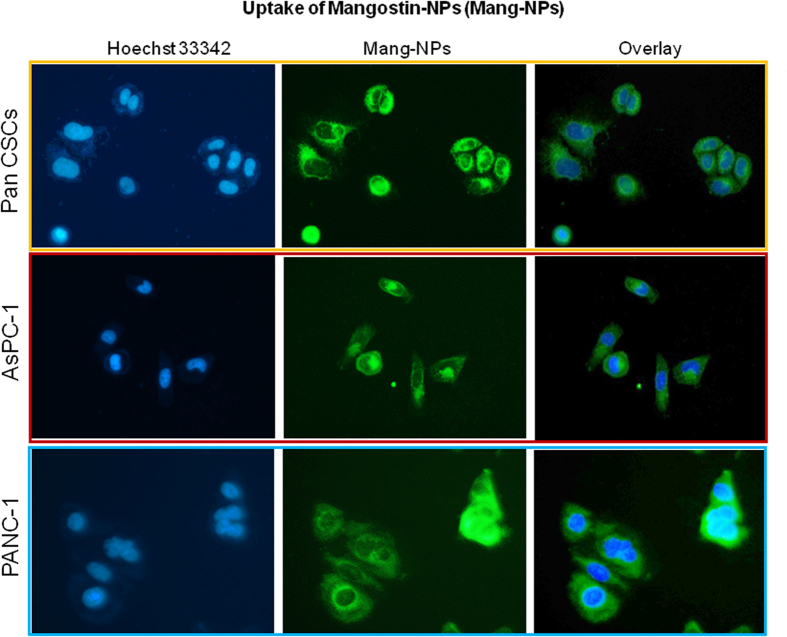
Uptake of Mang-NPs by Pancreatic cancer stem cells and cancer cell lines. Pancreatic CSCs, and cancer cell lines (AsPC-1 and PANC-1) were treated with coumarin-6 containing Mang-NPs for 2 h. Cells were incubated with Hoechst 33342 for nuclear staining. The fluorescence microscope was used to observe the uptake of Mang-NPs. Green color = Mang-NP. Blue color = Nucleus.

PLGA (50:50 PLGA, 14000–16000 MW, Sigma-Aldrich) nanoparticles (NPs) encapsulating α-mangostin and coumarin-6 (fluorescent dye) were prepared using a double emulsion-solvent evaporation method. Mang-NPs possess the following physico-chemical characteristics: % PVA = 0.50 (w/v); Particle Size = 186.3 ± 6.42 nm; Zeta Potential = 0.03 ± 0.005 mV; Drug encapsulation = 51.16 ± 2.61%. We have recently isolated CSCs from the pancreata of human and Kras^G12D^ mice^28^,^31^,^32^. We next examined the uptake of Mang-NPs by pancreatic CSCs and cancer cell lines (AsPC-1 and PANC-1). As shown in Fig. 1, pancreatic CSCs and cell lines were able to absorb Mang-NPs in 30 min as demonstrated by the green color. We have also observed the uptake of Mang-NPs as early as 10 min with a maximum fluorescence reaching at 30 min *in vitro*. These data suggest that Mang-NPs are functional and can easily enter into the cells.

### Mang-NPs inhibit cell proliferation of Pan CSCs and cell lines, but have no effect on human pancreatic normal ductal epithelial (HPNE) cells

**Figure 2:**
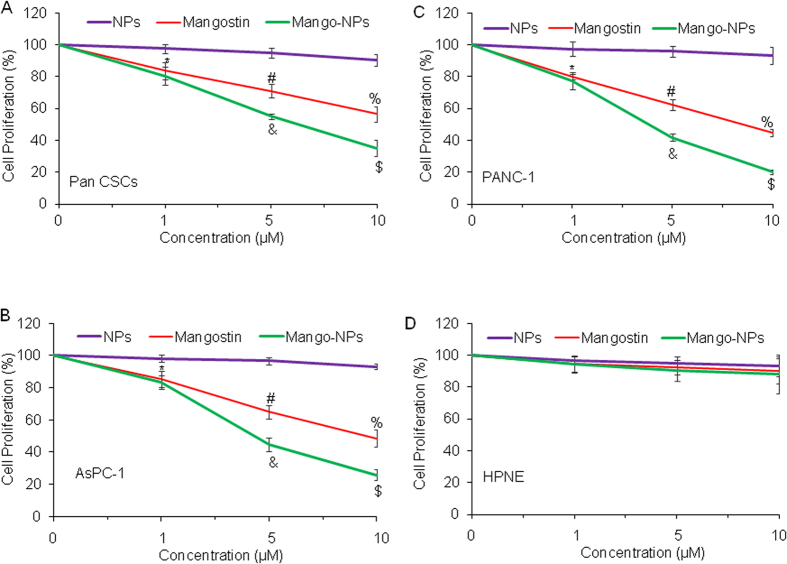
Mang-NPs inhibit cell proliferation of pancreatic CSCs and cancer cell lines. (**A**) Pancreatic CSCs were treated with PLGA-NPs (NPs), Mangostin or Mang-NPs (0–10 μM) for 48 hrs, and cell proliferation was measured. Data represent mean (n = 4) ± SD. *, #, &, % and $ = significantly different from control (NPs group), and each other, P < 0.05. (**B**) and (**C**) AsPC-1 and PANC-1 cells were treated with NPs, Mangostin or Mang-NPs (0–10 μM) for 48 hrs, and cell proliferation was measured. Data represent mean (n = 4) ± SD. *, #, &, % and $ = significantly different from control (NPs group), and each other, P < 0.05. (**D**) Human pancreatic normal ductal epithelial (HPNE) cells were treated with NPs, Mangostin or Mang-NPs (0–10 μM) for 48 hrs, and cell proliferation was measured. Data represent mean (n = 4) ± SD. Mango-NPs = Mang-NPs

We next examined the effects Mang-NPs on cell proliferation of Pancreatic CSCs, cell lines (PANC-1 and AsPC-1) and HPNE cells. Mangostin and Mang-NPs inhibited cell proliferation of Pan CSCs and cancer cell lines (AsPC-1 and PANC-1) in a dose-dependent manner (Fig. 2A–C). Mang-NPs were more effective than Mangostin (5 and 10 μM) in inhibiting cell proliferation of Pan CSCs and pancreatic cancer cell lines. By comparison, PLGA-NPs (NPs) had no effect on cell proliferation of CSCs and cancer cells. Interestingly, HPNE cells were not significantly affected by NPs, Mangostin and Mang-NPs (Fig. 2D). These data suggest that Mang-NPs can be used for the treatment of pancreatic cancer.

### Mang-NPs inhibit colony formation and induce apoptosis in pancreatic CSCs and cell lines

**Figure 3:**
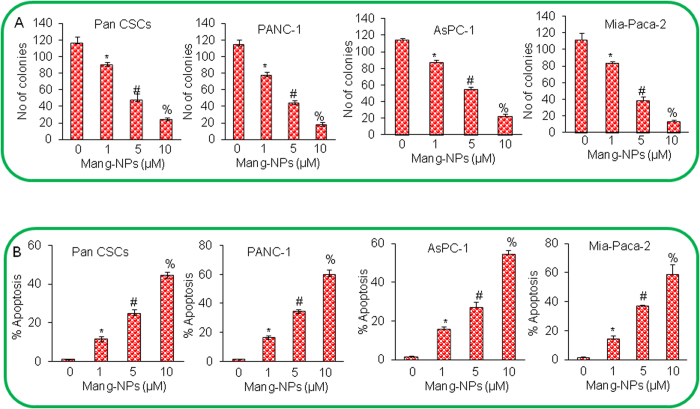
Mang-NPs inhibit colony formation and induce apoptosis in pancreatic CSCs and cancer cell lines. (**A**) Pancreatic CSCs (Pan CSCs), and cancer cell lines (PANC-1, AsPC-1, and MIA PaCa-2) were treated with Mang-NPs (0–10 μM) for 21 days. Number of colonies were counted. Data represent mean ± SD. *, # and % = significantly different from control, and each other, P < 0.05. (**B**) Pan CSCs, and cancer cell lines (PANC-1, AsPC-1, and MIA PaCa-2) were treated with Mang-NPs (0–10 μM) for 48 hrs. Apoptosis was measured by TUNEL assay. Data represent mean ± SD. *, # and % = significantly different from control, and each other, P < 0.05.

Colony formation and apoptosis induction are commonly used to assess the effectiveness of anticancer drugs. We next examined the effects of Mang-NPs on colony formation and apoptosis. Mang-NPs inhibited colony formation in Pan CSCs and cancer cell lines (PANC-1, AsPC-1 and MIA PaCa-2) in a dose-dependent manner (Fig. 3A). Similarly, Mang-NPs induced apoptosis in Pan CSCs and pancreatic cancer PANC-1, AsPC-1 and MIA PaCa-2 cell lines (Fig. 3B). These data suggest that Mang-NPs are effective in inhibiting both pancreatic CSCs and cell lines.

### Mang-NPs inhibit cell viability in spheroids formed by pancreatic CSCs isolated from humans and Kras^G12D^ mice

**Figure 4:**
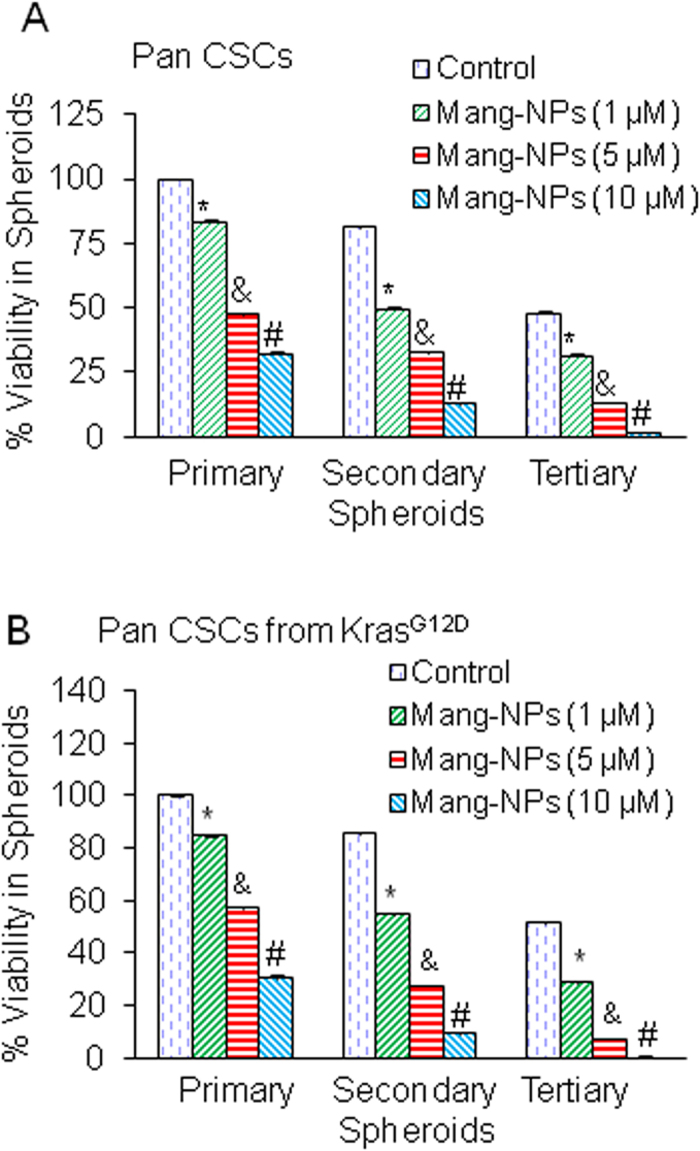
Mang-NPs inhibit cell viability in spheroids formed by Pan CSCs isolated from human and Kras^G12D^ mice. (**A**) Human pancreatic CSCs were treated with Mang-NPs (0–10 μM) for 7 days to obtain primary spheroids. At the end of incubation period, spheroids were collected, reseeded and treated with Mang-NPs for another week to obtain secondary spheroids. Secondary spheroids were collected, reseeded and treated with Mang-NPs for another week to obtain tertiary spheroids. Cell viability in spheroids was measured by trypan blue assay at the end of 7, 14 and 21 days. Data represent mean ± SD. *, & and # = significantly different from control, P < 0.05. (**B**) Mouse pancreatic CSCs were treated with Mang-NPs (0–10 μM) for 7 days to obtain primary spheroids. At the end of incubation period, spheroids were collected, reseeded and treated with Mang-NPs for another week to obtain secondary spheroids. Secondary spheroids were collected, reseeded and treated with Mang-NPs for another week to obtain tertiary spheroids. Cell viability in spheroids was measured by trypan blue assay at the end of 7, 14 and 21 days. Data represent mean ± SD. *, & and # = significantly different from control, P < 0.05.

Since CSCs play a major role in cancer initiation, progression, metastasis and drug resistance, they can be used to assess the response of anticancer drugs. Spheroid formation in suspension is one of the characteristics of CSCs. Kras^G12D^ mice mimic pancreatic cancer development in humans. Pancreatic CSCs isolated from Kras^G12D^ mice are phenotypically similar to pancreatic CSCs isolated from humans. We next examined the effects of Mang-NPs on growth of pancreatic CSCs isolated from humans and Kras^G12D^ mice by measuring cell viability in spheroids. Mang-NPs inhibited cell viability of primary, secondary and tertiary spheroids formed by pancreatic CSCs isolated from human and Kras^G12D^ mice (Fig. 4A,B). These data suggest that Mang-NPs can be used for the management of pancreatic cancer by targeting pancreatic CSCs.

### Mang-NPs inhibit cell motility, migration and invasion and markers of epithelial-mesenchymal transition

**Figure 5:**
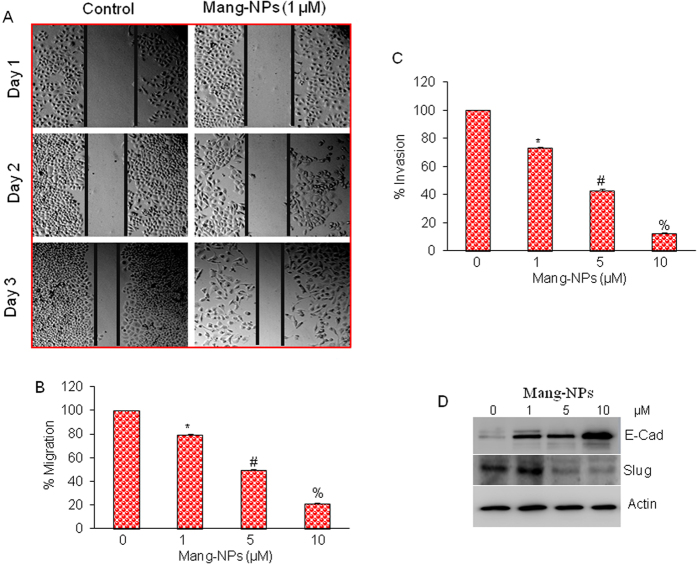
α-mangostin inhibits cell motility, migration and invasion and modulates the expression markers of epithelial-mesenchymal transition (EMT). (**A**) Pan CSCs were grown in monolayer, scratched and treated with or without Mang-NPs (0–1 μM) for 1 or 2 days. Cells were photographed as we described elsewhere^22^,^50^. (**B**,**C**) Cell Migration and invasion assay. Pan CSCs were seeded, treated with Mang-NPs (0–10 μM) for 48 h and cell migration and invasion assays were performed as described in Materials and Methods. Data represent mean (n = 4) ± SD. *, #, and % = significantly different from control, and each other, P < 0.05. (**D**) Pan CSCs were treated with Mang-NPs (0–10 μM) for 48 hrs. The expression of E-cadherin, and Slug was measured by the Western blot analysis. β-actin was used as an internal control.

Epithelial-mesenchymal transition (EMT) is a biological process by which cells undergo through genetic changes that allow them to leave the primary site and migrate to distant location (secondary site) to reestablish and proliferate^33^. Since CSCs have been shown to be the cause of metastasis, we next measured the effects of Mang-NPs on cell motility, migration, and invasion, and on the expression of EMT markers. Mang-NPs inhibited cell motility, migration, and invasion of Pan CSCs (Fig. 5A–C). Similarly, Mang-NPs inhibited cell motility, migration and invasion in pancreatic cancer AsPC-1 and PANC-1 cell lines (data not shown).

Since Mang-NPs inhibited cell motility, migration, and invasion, we next examined the molecular mechanisms of EMT regulation by measuring the expression of proteins which modulate EMT, i.e. E-cadherin, N-cadherin and transcription factor Slug. As shown in Fig. 5D, Mang-NPs induced the expression of E-cadherin, and inhibited the expression of Slug. These data suggest that Mang-NPs has the potential to inhibit EMT by modulating the expression of E-cadherin and Slug.

### Mang-NPs inhibit pluripotency maintaining factors in pancreatic CSCs

**Figure 6:**
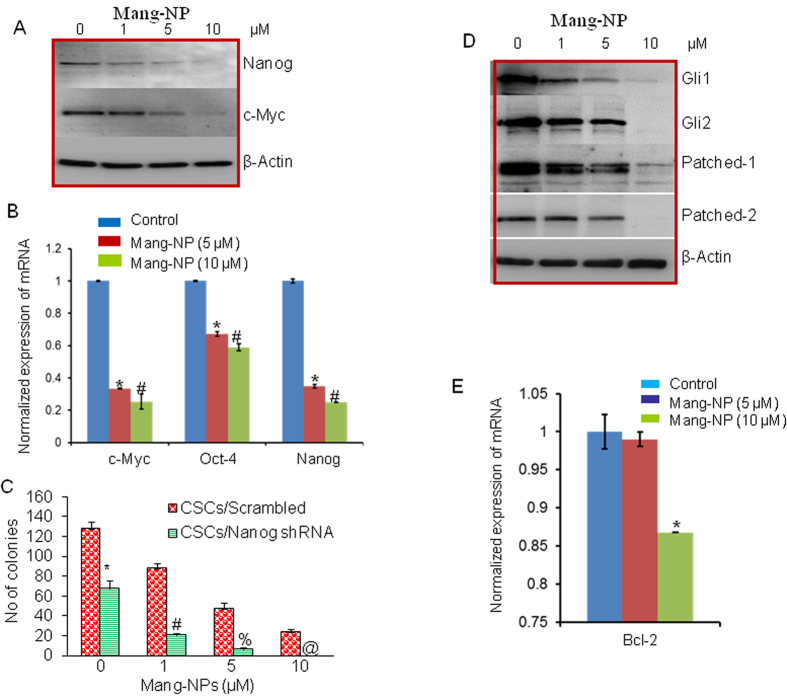
Regulation of pluripotency maintaining factors, Shh pathway, and Bcl-2 by Mang-NPs. (**A**) Pancreatic CSCs were treated with Mang-NPs (0–10 μM) for 48 h. The expression of Nanog, and c-Myc was measured by the Western blot analysis. β-actin was used as a loading control. (**B**) Pancreatic CSCs were treated with Mang-NPs (0–10 μM) for 36 h. The expression of c-Myc, Oct-4 and Nanog was measured by q-RT-PCR. Data represent mean ± SD. * and # = significantly different from control, P < 0.05. (**C**) Nanog shRNA enhances the inhibitory effects of Mang-NPs on colony formation. Pan CSCs/Scrambled and CSCs/Nanog shRNA were seeded and treated with Mang-NPs (0–10 μM) for 21 days. At the end of incubation period, number of colonies were counted. Data represent mean ± SD. *, #, %, and @ = significantly different from control, P < 0.05. (**D**) Pancreatic CSCs were treated with Mang-NPs (0–10 μM) for 48 h. The expression of Gli1, Gli2, Patched-1, and Patched-2 was measured by the Western blot analysis. β-actin was used as a loading control. (**E**), Pancreatic CSCs were treated with Mang-NPs (0–10 μM) for 36 h. The expression of Bcl-2 was measured by q-RT-PCR. Data represent mean ± SD. * = significantly different from control, P < 0.05.

Nanog, and c-Myc are essential for maintaining pluripotency in pancreatic CSCs. We therefore examined the effects of Mang-NPs on the expression of Nanog and c-Myc. Pancreatic CSCs were treated with Mang-NPs and the expression of Nanog and c-Myc was measured by the Western blot analysis. As shown in Fig. 6A, Mang-NPs inhibited the expression of Nanog in a dose dependent manner. In addition, while 1 μM of Mang-NPs was ineffective in inhibiting c-Myc expression, higher doses of Mang-NPs (5 and 10 μM) significantly inhibited c-Myc expression in Pan CSCs. We next confirmed the expression of these pluripotency maintaining factors by q-RT-PCR. Mang-NPs inhibited the expression of c-Myc, Oct4 and Nanog (Fig. 6B). These data suggest that Mang-NPs can inhibit CSC characteristics by inhibiting the expression of pluripotency maintain factors.

Nanog is required for maintaining pluripotency and self-renewal of CSCs. We therefore examined whether inhibition of Nanog by shRNA can enhance the anticancer activity of Mang-NPs. Pan CSCs were transduced with lentiviral particles expressing either scrambled or Nanog shRNA. Transduced CSCs were treated with Mang-NPs to examine their effects on colony formation. Pan CSCs/Nanog shRNA group had significantly lower number of colonies than Pan CSCs/Scrambled group (Fig. 6C). Furthermore, Mang-NPs inhibited colony formation in Pan CSCs/Scrambled group in a dose-dependent manner. Interestingly, the inhibitory effects of Mang-NPs were significantly higher in Pan CSCs/Nanog shRNA group than Pan CSCs/Scrambled group at all the doses used. These data demonstrate that inhibition of Nanog expression may be a novel strategy to enhance the effectiveness of Mang-NPs.

### Mang-NPs inhibit Shh signaling pathway and Gli transcriptional target proteins

Aberrant activation of Shh signaling pathway has been demonstrated in pancreatic cancer^5^,^34^. We next examined the effects of Mang-NPs on components of SHH pathway by measuring the expression of Gli1, Gli2, Patched 1, and Patched 2 by Western blot analysis. Mang-NPs inhibited the expression of Gli1, Gli2, Patched 1 and Patched 2 (Fig. 6D). Inhibition of Gli regulates its own expression and also the expression of Patched because they are the direct target of transcription factor Gli. These data suggest that Mang-NPs can inhibit self-renewal capacity of pancreatic CSCs by targeting Shh pathway.

Bcl-2 plays a major role in cell survival and also inhibits apoptosis. Further, it is a direct transcriptional target of Gli. We therefore sought to examine whether Mang-NPs can regulate the expression of Bcl-2 in Pan CSCs (Fig. 6E). Low doses of Mang-NPs (up to 5 μM) had no effect on Bcl-2 expression, probably because the expression of Gli2 was also not affected at the similar doses of Mang-NPs. However, 10 μM dose of Mang-NPs inhibited the expression of Bcl-2. These data suggest that Bcl-2 can modulate the anti-proliferative activity of Mang-NPs.

**Figure 7:**
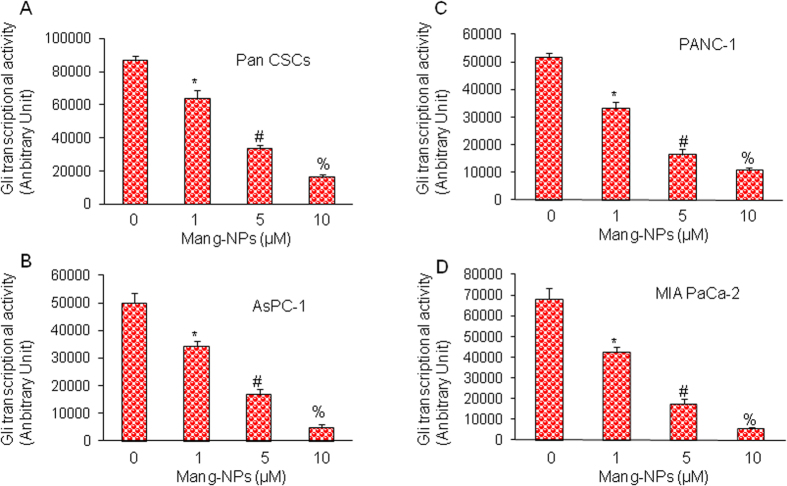
Regulation of Gli transcription by Mang-NPs. (**A**–**D**) Pancreatic CSCs, and cancer cell lines (PANC-1, AsPC-1, and MIA PaCa-2) were stably transduced with Gli-responsive GFP/firefly luciferase viral particles (pGreen Fire1-Gli with EF1, System Biosciences). Transduced CSCs and cell lines were treated with Mang-NPs (0–10 μM) for 24 h. Gli reporter activity was measured as we described^25^. Data represent mean ± SD. *, # and % = significantly different from control, P < 0.05.

We next measured the effects of Mang-NPs on Gli transcriptional activity in Pan CSCs, and cancer cell lines (PANC-1, AsPC-1 and MIA PaCa-2) by luciferase reporter assay. Mang-NPs inhibited Gli reporter activity in Pan CSCs and pancreatic cancer cell lines in a dose-dependent manner (Fig. 7A–D). These data suggest that Mang-NPs can inhibit cell proliferation of Pan CSCs and cancer cell lines by inhibiting Shh-Gli pathway.

### Mang-NPs inhibit pancreatic cancer growth, development and metastasis in KPC (Pdx^Cre^;LSLKras^G12D^;LSL-Trp53^R172H^) mice

**Figure 8:**
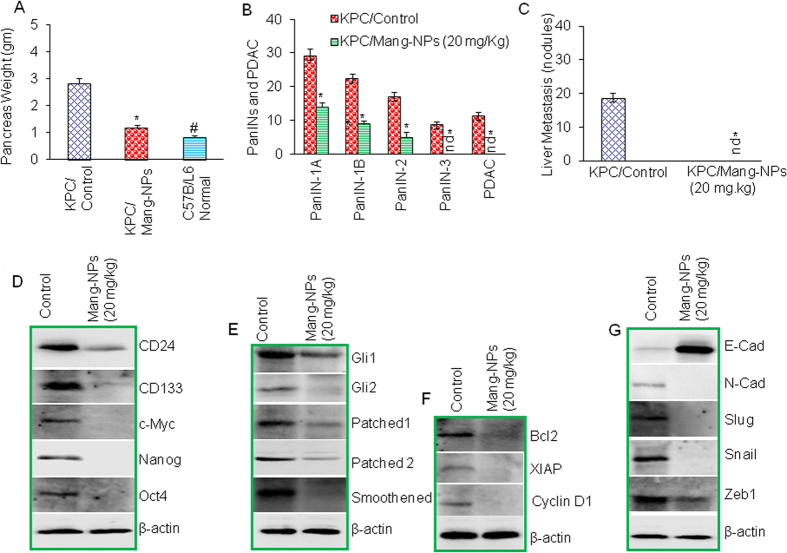
Mang-NPs inhibits pancreatic cancer growth in KPC mice by suppressing Shh-Gli pathway, and modulating the expression of stem cells markers, pluripotency maintaining factor, EMT markers and Bcl-2, XIAP and Cyclin D1. (**A**) Inhibition of pancreatic cancer growth and development in KPC mice. KPC mice were treated with Mang-NPs (20 mg/kg) for about 10 weeks. At the end of experiment, mice were sacrificed and pancreas weight was recorded. Data represent mean ± SD. * and # = significantly different from untreated control group, P < 0.05. (**B**) Pancreatic tissues from control and Mang-NPs-treated mice were fixed and stained with H & E. Tissue sections were visualized for the presence of PanIN (PanIN-1A, PanIN-1B, PanIN-2, and PanIN-3) lesions and PDAC. nd = not detected. (**C**) Liver metastasis. Liver tissues from control and Mang-NPs-treated mice were visualized for the presence of nodules. (**D**) Expression of stem cell marker, and pluripotency maintain factors in tumor tissues. Pancreatic cancer tissues from control and Mang-NPs treated mice were subjected to the Western blot analysis, and the expression of CD24, CD133, c-Myc, Nanog and Oct4 was measured. β-actin was used as a loading control. (**E**) Expression of components of Shh pathway in tumor tissues. Pancreatic cancer tissues from control and Mang-NPs treated mice were subjected to the Western blot analysis, and the expression of Gli1, Gli2, Patched1, Patched2, and Smoothened was measured. (**F**) Expression of Bcl2, XIAP and Cyclin D1 in tumor tissues. Pancreatic cancer tissues from control and Mang-NPs treated mice were subjected to the Western blot analysis, and the expression of Bcl2, XIAP and Cyclin D1 was measured. (**G**) Expression of E-cadherin, N-Cadherin, Slug, Snail, and Zeb1 in tumor tissues. Pancreatic cancer tissues from control and Mang-NPs treated mice were subjected to the Western blot analysis, and the expression of E-cadherin, N-Cadherin, Slug, Snail, and Zeb1 was measured.

KPC (Pdx^Cre^;LSLKras^G12D^;LSL-Trp53^R172H^) mice mimics the pancreatic cancer development in humans and have been used to examine the effects of anticancer drugs. We therefore used KPC mice to examine whether Mang-NPs regulate pancreatic cancer growth, development and metastasis. The treatment of Mang-NPs was started when mice were about 4 weeks old. Mang-NPs were administrated intraperitoneally once per day, five days per week for about 10 weeks. We first measured the weight of pancreas from KPC/control, KPC/Mang-NPs and C57B/L6 mice (untreated). The data demonstrate that KPC/Mang-NPs group had significantly lower pancreas weight than KPC/control group, and C57B/L6 normal group (Fig. 8A). The pancreas weight from KPC/Mang-NPs group was slightly but significantly higher than C57B/L6 mice (untreated). These data suggest that Mang-NPs can inhibit the growth of pancreatic cancer.

The specimens were evaluated for the presence of PanIN-1A, PanIN-1B, PanIN-2, and PanIN-3, and adenocarcinoma (PDAC). As shown in Fig. 8B, pancreata of KPC/Control group had PanIN-1A, PanIN-1B, PanIN-2, PanIN-3, and PDAC. All pancrerata with pancreatic adenocarcinoma had concomitant PanIN lesions. Treatment of KPC group with Mang-NPs resulted in significant inhibition of PanIN-1A, PanIN-1B, and PanIN-2 lesions and absence of PanIN-3, and PDAC. These data suggest that Mang-NPs can inhibit pancreatic cancer growth and progression.

Liver metastasis is very common in late stage of pancreatic cancer^2^. We therefore measured liver metastasis by counting the number of nodules in the liver in control and Mang-NPs groups (Fig. 8C). We have observed liver metastasis in KPC/control group. By comparison, KPC/Mang-NPs group showed no liver metastasis. Overall, these data suggest that Mang-NPs can inhibit pancreatic cancer growth, development and metastasis in KPC mice.

### Inhibition of pancreatic carcinogenesis by Mang-NPs was correlated with suppression of Shh-Gli pathway and its downstream targets, and modulation of protein expression of stem cells markers, pluripotency maintaining factor, and EMT markers

Since Mang-NPs inhibited growth, development and metastasis of pancreatic cancer in KPC mice, we next examined the molecular mechanisms of these pathological events. We have previously demonstrated the presence of CSCs in the pancreata of Kras^G12D^ and KPC mice^26^,^29^. Tumor tissues from control and Mang-NPs treated groups were isolated, and tissue lysates were subjected to the Western blot analysis. Since KPC mice have been shown to harbor a small population of stem cells, we first examined the effects of Mang-NPs on the expression of stem cell markers (CD24 and CD133) and pluripotency maintaining factors (c-Myc, Nanog and Oct4). Treatment of KPC mice with Mang-NPs resulted in downregulation of CD24, CD133, c-Myc, Nanog and Oct4 (Fig. 8D). These data suggest that Mang-NPs can inhibit stem cell population within the tumor.

Since Mang-NPs inhibited pancreatic CSC characteristics *in vitro* by suppressing Shh pathway, we next examined the effects of Mang-NPs on the components of Shh pathway and its downstream targets in tumor tissues derived from KPC mice. Mang-NPs downregulated the expression of Gli1, Gli2, Patched-1, Patched-2 and smoothened in KPC mice (Fig. 8E). Furthermore, Mang-NPs also inhibited the expression of Gli targets Bcl-2, XIAP and cyclin D1 (Fig. 8F). These data suggest that Mang-NPs can regulate pancreatic carcinogenesis by inhibiting Shh pathway and also its targets which control apoptosis (Bcl-2), cell survival (XIAP) and cell cycle (cyclin D1).

Since Mang-NPs inhibited EMT *in vitro* and liver metastasis *in vivo*, we sought to examine the effects of Mang-NPs on markers of EMT in tumor tissues. As shown in Fig. 8G, Mang-NPs induced the expression of E-cadherin and inhibited the expression of N-cadherin, Slug, Snail, and Zeb1. These data suggest that Mang-NPs can reverse EMT by causing cadherin switch and inhibiting the EMT transcription factor.

## Discussion

In this paper, we have demonstrated for, the first time, that Mang-NPs can inhibit pancreatic CSC characteristics, tumor growth and metastasis. Specifically, (i) Mang-NPs can be easily uptaken by the Panc CSCs and cancer cell lines; (ii) Mang-NPs inhibit cell viability of pancreatic CSCs and cell lines without inducing any toxicity to human normal pancreatic ductal epithelial cells; (iii) Mang-NPs inhibit colony formation and spheroid formation, and induce apoptosis in pan CSCs; (iv) Mang-NPs inhibit EMT by inducing cadherin switch and suppressing the expression of EMT transcription factors; (v) Mang-NPs inhibit self-renewal capacity of Pan CSC by suppressing pluripotency maintaining factors, and components of Shh pathway and its downstream targets; and (vi) Mang-NPs inhibit pancreatic cancer growth, development and metastasis in KPC mice by modulating the expression of stem cell and EMT markers, pluripotency maintain factors, and cell survival, apoptosis and cell cycle proteins. The ability of Mang-NPs to inhibit pancreatic carcinogenesis and CSC characteristics suggests that it not only inhibits bulk tumor mass but also CSCs which have been shown to be the cause of cancer initiation, progression and metastasis.

Kras^G12D^ mutations are very common in pancreatic cancer, and more than 90% pancreatic cancer patients harbor mutations in this gene^35^. There are no effective drugs which have been designed to treat pancreatic cancer by targeting Kras^G12D^. In our study, Mang-NPs inhibited cell viability of spheroids formed by CSCs isolated from humans and Kras^G12D^ mice, and also inhibit cell proliferation and induce apoptosis in pancreatic cancer cell lines harboring Kras^G12D^ mutations. Furthermore, Mang-NPs also inhibited PanIN formation, PDAC development and metastasis in KPC mice, suggesting Mang-NPs can be a useful drug for the treatment and/or prevention of pancreatic cancer which harbor CSCs and progenitor cells.

Aberrant activation of Shh signaling pathway has been implicated in several cancers such as pancreatic cancer, prostate, glioblastoma, squamous cell carcinoma, basal cell carcinoma, medulloblastoma, prostate cancer, colon cancer, rhabdomyosarcoma and breast cancer^31^,^36^,^37^,^38^,^39^,^40^. We have recently demonstrated that various components of Shh signaling pathway including Gli1, Gli2, Patched-1, Patched-2, and Smoothened are expressed in Pan CSCs and pancreatic cancer cell lines^20^,^21^,^23^,^25^,^31^,^41^. Gli, Patched, smoothened, cyclin D1 and Bcl-2 are the transcriptional targets of Gli. In the present study, we have demonstrated that Mang-NPs can inhibit various components of the Shh pathway and its targets such as Bcl2, XIAP and Cyclin D1. These data suggest that Mang-NPs can inhibit pancreatic carcinogenesis by inhibiting cell survival, proliferation and cell cycle through Shh pathway. Similarly, in another study we have demonstrated that anthothecol-encapsulated PLGA nanoparticles can inhibit pancreatic CSC characteristics by inhibiting Shh pathway^26^.

EMT is an early pathological event which is characterized by the activation of embryonic programs of epithelial plasticity and switch from a sessile, epithelial phenotype to a motile, mesenchymal phenotype^42^. During early events, the expression of E-cadherin (CDH1 gene) goes down and the expression of N-cadherin goes up due to activation of transcription factor Snail, Slug and Zeb1^43^. CSCs also follow the similar patterns of cellular movement for sustained metastatic growth where the dissemination of CSCs from the primary tumor site is followed by their re-establishment in a secondary site. Thus, EMT can be considered as an early event during metastasis. In our *in vitro* studies, 1 μM dose (a low dose) of Mang-NPs inhibited cell motility, invasion and migration, but had no effect on c-Myc expression. It could be due to the fact that Mang-NPs inhibited Nanog which can inhibit cell motility, invasion and migration. Our previous and present studies establish a key and essential role of the Shh-Gli pathway in promoting pancreatic CSC tumor growth, stem cell self-renewal and metastatic behavior^20^,^23^,^25^,^29^,^31^,^44^,^45^. In the present study, Mang-NPs not only inhibited tumor growth and development (PanINs to PDAC) but also metastasis to liver.

Nanotechnology has been successfully used to deliver anticancer drugs because nanoparticles enhance the bioavailability and distribution, preserve the integrity of compounds, and improve the biological activity of drugs. In the present study, Mang-NPs inhibited pancreatic cancer cell growth and CSC characteristics *in vitro*, and tumor growth, development and metastasis in KPC mice. Similarly, the clinical significance of α-Mangostin or Garcinia mangostana extract containing nanoparticles using different carriers have been reported in cancer and other diseases. α-Mangostin encapsulated Gold/polyethyleneimine/cyclodextrin (AuNPs/PEI/CD) nanoparticles inhibited prostate cancer PC-3 and DU-145 cell viability^46^. Furthermore, α-Mangostin encapsulated poly(ethylene glycol)-poly(l-lactide) (PEG-PLA) nanoparticles ameliorates Alzheimer’s disease neuropathology by elevating low density lipoprotein receptor (LDLR) expression in microglia and liver cells and accelerating amyloid-beta clearance^47^. A clinical trial with the 4-week-randomized, double-blind, placebo-controlled, split-face study in 10 acne patients demonstrated a significant improvement in acne vulgaris condition with cellulose-based α-mangostin nanoparticles^48^. In another study, oral administration of mucoadhesive nanoparticles loaded with Garcinia mangostana extract eradicated Helicobacter pylori infection in mice^49^.

In conclusion, our study has demonstrated for the first time that Mang-NPs can inhibit proliferation of pancreatic CSCs and cancer cell lines, and inhibit the self-renewal capacity of CSCs isolated from pancreata of human and Kras^G12D^ mice. Mang-NPs also inhibited pancreatic cancer progression from PanINs to PDAC and metastasis in KPC mice by suppressing Shh pathway. Since Mang-NPs inhibited the expression of Pan CSC markers and pluripotency maintaining factors in pancreatic tumors, it suggests that Mang-NPs can inhibit carcinogenesis by targeting CSC population. Overall these data suggest that Mang-NPs offer new hope for the treatment and/or prevention of pancreatic cancer by targeting CSCs. Based on our novel findings, clinical trials are needed to validate the role of Mang-NPs for the treatment and/or prevention of pancreatic cancer.

## Additional Information

**How to cite this article**: Verma, R. K. *et al*. α-Mangostin-encapsulated PLGA nanoparticles inhibit pancreatic carcinogenesis by targeting cancer stem cells in human, and transgenic (Kras^G12D^, and Kras^G12D^/tp53R270H) mice. *Sci. Rep.* **6**, 32743; doi: 10.1038/srep32743 (2016).

## References

[b1] Siegel R. L., Miller K. D. & Jemal A. Cancer statistics, 2015. CA Cancer J Clin 65, 5–29, 10.3322/caac.21254 (2015). 25559415

[b2] Singh D., Upadhyay G., Srivastava R. K. & Shankar S. Recent advances in pancreatic cancer: biology, treatment, and prevention. Biochim Biophys Acta 1856, 13–27, 10.1016/j.bbcan.2015.04.003 (2015). 25977074

[b3] Babu V., Paul N. & Yu R. Animal models and cell lines of pancreatic neuroendocrine tumors. Pancreas 42, 912–923, 10.1097/MPA.0b013e31827ae993 (2013). 23851429

[b4] Morton J. P. & Lewis B. C. Shh signaling and pancreatic cancer: implications for therapy? Cell Cycle 6, 1553–1557, 4467 [pii] (2007). 17611415 10.4161/cc.6.13.4467

[b5] Thayer S. P. . Hedgehog is an early and late mediator of pancreatic cancer tumorigenesis. Nature 425, 851–856 (2003). 14520413 10.1038/nature02009 PMC3688051

[b6] Ee G. C., Daud S., Taufiq-Yap Y. H., Ismail N. H. & Rahmani M. Xanthones from Garcinia mangostana (Guttiferae). Nat Prod Res 20, 1067–1073, 10.1080/14786410500463114 (2006). 17127660

[b7] Ji X., Avula B. & Khan I. A. Quantitative and qualitative determination of six xanthones in Garcinia mangostana L. by LC-PDA and LC-ESI-MS. J Pharm Biomed Anal 43, 1270–1276, 10.1016/j.jpba.2006.10.018 (2007). 17129697

[b8] Marcason W. What are the facts and myths about mangosteen? J Am Diet Assoc 106, 986, 10.1016/j.jada.2006.04.008 (2006). 16720137

[b9] Hafeez B. B. . alpha-Mangostin: a dietary antioxidant derived from the pericarp of Garcinia mangostana L. inhibits pancreatic tumor growth in xenograft mouse model. Antioxid Redox Signal 21, 682–699, 10.1089/ars.2013.5212 (2014). 24295217 PMC4104617

[b10] Jittiporn K. . Anti-angiogenic actions of the mangosteen polyphenolic xanthone derivative alpha-mangostin. Microvasc Res 93, 72–79, 10.1016/j.mvr.2014.03.005 (2014). 24721607 PMC4075264

[b11] Kritsanawong S., Innajak S., Imoto M. & Watanapokasin R. Antiproliferative and apoptosis induction of alpha-mangostin in T47D breast cancer cells. Int J Oncol 48, 2155–2165, 10.3892/ijo.2016.3399 (2016). 26892433

[b12] Shan T. . alpha-Mangostin suppresses human gastric adenocarcinoma cells *in vitro* via blockade of Stat3 signaling pathway. Acta Pharmacol Sin 35, 1065–1073, 10.1038/aps.2014.43 (2014). 24976157 PMC4125713

[b13] Xu Q. . Alpha-Mangostin suppresses the viability and epithelial-mesenchymal transition of pancreatic cancer cells by downregulating the PI3K/Akt pathway. Biomed Res Int 2014, 546353, 10.1155/2014/546353 (2014). 24812621 PMC4000937

[b14] Mizushina Y., Kuriyama I., Nakahara T., Kawashima Y. & Yoshida H. Inhibitory effects of alpha-mangostin on mammalian DNA polymerase, topoisomerase, and human cancer cell proliferation. Food Chem Toxicol 59, 793–800, 10.1016/j.fct.2013.06.027 (2013). 23811100

[b15] Ma Y. . α-Mangostin inhibits pancreatic cancer stem cell characteristics in human and KrasG12D mice by modulating pluripotency maintaining factors and epithelial-mesenchymal transition: Involvement of Nanog and Sonic hedgehog pathway. Inter J Mol Sci (2016).

[b16] Varjosalo M. & Taipale J. Hedgehog: functions and mechanisms. Genes Dev 22, 2454–2472, 22/18/2454 [pii] 10.1101/gad.1693608 (2008). 18794343

[b17] Beachy P. A., Karhadkar S. S. & Berman D. M. Tissue repair and stem cell renewal in carcinogenesis. Nature 432, 324–331, nature03100 [pii] 10.1038/nature03100 (2004). 15549094

[b18] Rohatgi R., Milenkovic L. & Scott M. P. Patched1 regulates hedgehog signaling at the primary cilium. Science 317, 372–376, 317/5836/372 [pii] 10.1126/science.1139740 (2007). 17641202

[b19] Osterlund T. & Kogerman P. Hedgehog signalling: how to get from Smo to Ci and Gli. Trends Cell Biol 16, 176-180, S0962-8924(06)00053-5 [pii] 10.1016/j.tcb.2006.02.004 (2006). 16516476

[b20] Fu J. . GANT-61 inhibits pancreatic cancer stem cell growth *in vitro* and in NOD/SCID/IL2R gamma null mice xenograft. Cancer Lett 330, 22–32, 10.1016/j.canlet.2012.11.018 (2013). 23200667 PMC4153855

[b21] Huang M. . Embelin suppresses growth of human pancreatic cancer xenografts, and pancreatic cancer cells isolated from KrasG12D mice by inhibiting Akt and Sonic hedgehog pathways. Plos One 9, e92161, 10.1371/journal.pone.0092161 (2014). 24694877 PMC3973629

[b22] Li S. H., Fu J., Watkins D. N., Srivastava R. K. & Shankar S. Sulforaphane regulates self-renewal of pancreatic cancer stem cells through the modulation of Sonic hedgehog-GLI pathway. Mol Cell Biochem 373, 217–227, 10.1007/s11010-012-1493-6 (2013). 23129257

[b23] Marsh J. L., Jackman C. P., Tang S. N., Shankar S. & Srivastava R. K. Embelin suppresses pancreatic cancer growth by modulating tumor immune microenvironment. Front Biosci (Landmark Ed.) 19, 113–125 (2014). 24389175 10.2741/4198

[b24] Quint K. . The expression pattern of PDX-1, SHH, Patched and Gli-1 is associated with pathological and clinical features in human pancreatic cancer. Pancreatology 9, 116–126, 000178882 [pii] 10.1159/000178882 (2009). 19077462

[b25] Tang S. N. . Inhibition of sonic hedgehog pathway and pluripotency maintaining factors regulate human pancreatic cancer stem cell characteristics. Int J Cancer 131, 30–40, 10.1002/ijc.26323 (2012). 21796625 PMC3480310

[b26] Verma R. K., Yu W., Singh S. P., Shankar S. & Srivastava R. K. Anthothecol-encapsulated PLGA nanoparticles inhibit pancreatic cancer stem cell growth by modulating sonic hedgehog pathway. Nanomedicine 11, 2061–2070, 10.1016/j.nano.2015.07.001 (2015). 26199979

[b27] Hingorani S. R. Location, location, location: precursors and prognoses for pancreatic cancer. Gastroenterology 133, 345–350 (2007). 17631154 10.1053/j.gastro.2007.05.059

[b28] Shankar S. . Resveratrol inhibits pancreatic cancer stem cell characteristics in human and KrasG12D transgenic mice by inhibiting pluripotency maintaining factors and epithelial-mesenchymal transition. Plos One 6, e16530, 10.1371/journal.pone.0016530 (2011). 21304978 PMC3031576

[b29] Sharma N. . PI3K/AKT/mTOR and sonic hedgehog pathways cooperate together to inhibit human pancreatic cancer stem cell characteristics and tumor growth. Oncotarget 6, 32039–32060, 10.18632/oncotarget.5055 (2015). 26451606 PMC4741658

[b30] Hingorani S. R. . Trp53R172H and KrasG12D cooperate to promote chromosomal instability and widely metastatic pancreatic ductal adenocarcinoma in mice. Cancer Cell 7, 469–483 (2005). 15894267 10.1016/j.ccr.2005.04.023

[b31] Singh B. N., Fu J., Srivastava R. K. & Shankar S. Hedgehog signaling antagonist GDC-0449 (Vismodegib) inhibits pancreatic cancer stem cell characteristics: molecular mechanisms. Plos One 6, e27306, 10.1371/journal.pone.0027306 (2011). 22087285 PMC3210776

[b32] Nalls D., Tang S. N., Rodova M., Srivastava R. K. & Shankar S. Targeting epigenetic regulation of miR-34a for treatment of pancreatic cancer by inhibition of pancreatic cancer stem cells. Plos One 6, e24099, 10.1371/journal.pone.0024099 (2011). 21909380 PMC3166078

[b33] Hanahan D. & Weinberg R. A. Hallmarks of cancer: the next generation. Cell 144, 646–674, 10.1016/j.cell.2011.02.013 (2011). 21376230

[b34] Von Hoff D. D. . Inhibition of the hedgehog pathway in advanced basal-cell carcinoma. N Engl J Med 361, 1164–1172, NEJMoa0905360 [pii] 10.1056/NEJMoa0905360 (2009). 19726763

[b35] Park S. W. . Oncogenic KRAS induces progenitor cell expansion and malignant transformation in zebrafish exocrine pancreas. Gastroenterology 134, 2080–2090, S0016-5085(08)00419-8 [pii] 10.1053/j.gastro.2008.02.084 (2008). 18549880 PMC2654247

[b36] Chari N. S. & McDonnell T. J. The sonic hedgehog signaling network in development and neoplasia. Adv Anat Pathol 14, 344–352, 10.1097/PAP.0b013e3180ca8a1d00125480-200709000-00006 [pii] (2007). 17717435

[b37] Datta S. & Datta M. W. Sonic Hedgehog signaling in advanced prostate cancer. Cell Mol Life Sci 63, 435–448, 10.1007/s00018-005-5389-4 (2006). 16389455 PMC11136125

[b38] Daya-Grosjean L. & Couve-Privat S. Sonic hedgehog signaling in basal cell carcinomas. Cancer Lett 225, 181–192, S0304-3835(04)00780-3 [pii] 10.1016/j.canlet.2004.10.003 (2005). 15978322

[b39] van den Brink G. R. Hedgehog signaling in development and homeostasis of the gastrointestinal tract. Physiol Rev 87, 1343–1375, 87/4/1343 [pii] 10.1152/physrev.00054.2006 (2007). 17928586

[b40] Yang Z. J. . Medulloblastoma can be initiated by deletion of Patched in lineage-restricted progenitors or stem cells. Cancer Cell 14, 135–145, S1535-6108(08)00229-8 [pii] 10.1016/j.ccr.2008.07.003 (2008). 18691548 PMC2538687

[b41] Huang M. . Rottlerin suppresses growth of human pancreatic tumors in nude mice, and pancreatic cancer cells isolated from Kras(G12D) mice. Cancer Lett 353, 32–40, 10.1016/j.canlet.2014.06.021 (2014). 25050737

[b42] Thiery J. P., Acloque H., Huang R. Y. & Nieto M. A. Epithelial-mesenchymal transitions in development and disease. Cell 139, 871–890, S0092-8674(09)01419-6 [pii] 10.1016/j.cell.2009.11.007 (2009). 19945376

[b43] Iwatsuki M. . Epithelial-mesenchymal transition in cancer development and its clinical significance. Cancer Sci 101, 293–299, CAS1419 [pii] 10.1111/j.1349-7006.2009.01419.x (2010). 19961486 PMC11159985

[b44] Nanta R. . NVP-LDE-225 (Erismodegib) inhibits epithelial-mesenchymal transition and human prostate cancer stem cell growth in NOD/SCID IL2Rgamma null mice by regulating Bmi-1 and microRNA-128. Oncogenesis 2, e42, 10.1038/oncsis.2013.5 (2013). 23567619 PMC3641359

[b45] Fu J. . NPV-LDE-225 (Erismodegib) inhibits epithelial mesenchymal transition and self-renewal of glioblastoma initiating cells by regulating miR-21, miR-128, and miR-200. Neuro Oncol 15, 691–706, 10.1093/neuonc/not011 (2013). 23482671 PMC3661095

[b46] Qiu S. . Delivery of tanshinone IIA and alpha-mangostin from gold/PEI/cyclodextrin nanoparticle platform designed for prostate cancer chemotherapy. Bioorg Med Chem Lett, 10.1016/j.bmcl.2016.03.097 (2016). 27040657

[b47] Yao L. . Nanoformulated alpha-mangostin ameliorates Alzheimer’s disease neuropathology by elevating LDLR expression and accelerating amyloid-beta clearance. J Control Release 226, 1–14, 10.1016/j.jconrel.2016.01.055 (2016). 26836197

[b48] Pan-In P., Wongsomboon A., Kokpol C., Chaichanawongsaroj N. & Wanichwecharungruang S. Depositing alpha-mangostin nanoparticles to sebaceous gland area for acne treatment. J Pharmacol Sci 129, 226–232, 10.1016/j.jphs.2015.11.005 (2015). 26701606

[b49] Pan-in P. . Combating Helicobacter pylori infections with mucoadhesive nanoparticles loaded with Garcinia mangostana extract. Nanomedicine (Lond) 9, 457–468, 10.2217/nnm.13.30 (2014). 23731457

[b50] Roy S. K., Srivastava R. K. & Shankar S. Inhibition of PI3K/AKT and MAPK/ERK pathways causes activation of FOXO transcription factor, leading to cell cycle arrest and apoptosis in pancreatic cancer. J Mol Signal 5, 10, 1750-2187-5-10 [pii] 10.1186/1750-2187-5-10 (2010). 20642839 PMC2915986

